# Out‐of‐Equilibrium Supramolecular Assembly Sustained by Photocatalysis

**DOI:** 10.1002/anie.9082267

**Published:** 2026-03-10

**Authors:** E. Pelorosso, M. Scaccaglia, A. Fortunato, D. Alessi, F. Arcudi, A. Aliprandi

**Affiliations:** ^1^ Dipartimento Di Scienze Chimiche Università degli Studi di Padova Padova Italy

## Abstract

Some supramolecular structures persist because they are stable; others persist because they work. Here, we report a platinum(II) supramolecular system in which catalytic activity is uniquely associated with a far‐from‐equilibrium aggregate. The complex forms two emissive species: a kinetically trapped, orange‐emissive aggregate (**PtA**), and a thermodynamically stable, blue‐emissive aggregate (**PtB**). Only **PtA** is catalytically active, acting as a single‐component photocatalyst for hydrogen evolution in aqueous ascorbate without additional photosensitizers or co‐catalysts, whereas **PtB** is completely inactive.

Continuous irradiation drives and maintains **PtA** in an active, out‐of‐equilibrium state by coupling energy input directly to photocatalytic turnover, thereby preventing its conversion into the inactive thermodynamic **PtB** form. Upon removal of light, **PtA** irreversibly relaxes to **PtB**, and all photocatalytic activity is lost.

This system demonstrates how catalytic function can be confined to an out‐of‐equilibrium assembly and sustained only through ongoing energy dissipation.

## Graphical abstract

Catalysis sustains structure: A Pt(II) supramolecular photocatalyst persists in a far‐from‐equilibrium state only as long as it remains photocatalytically active. Without ongoing catalytic activity, the system irreversibly relaxes into a thermodynamically stable but inactive state (PtB). Catalysis is essential for sustaining its structure.

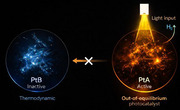

## 1 Introduction

Living systems rely on continuous energy input to sustain supramolecular architectures capable of performing highly specialized tasks [1, 2, 3]. These dynamic structures persist through constant energy dissipation that keeps them far from equilibrium, enabling functions that would be inaccessible in thermodynamic resting states [4, 5]. At its core, the required complexity for these systems arises not merely from molecular diversity but from dynamic organization—the ways in which molecules interact, assemble, and transform through self‐organization driven by non‐covalent interactions [6, 7].

Inspired by these principles, supramolecular chemistry has increasingly sought to harness controlled assembly pathways to endow artificial materials with emergent behaviors [8, 9, 10]. Aggregation can endow materials with novel collective properties such as aggregation‐induced emission (AIE) [11, 12, 13, 14], (semi)conductivity [15, 16, 17, 18, 19], or (photo)catalytic activity [20, 21, 22, 23], demonstrating how molecular packing and morphology, beyond mere molecular identity, dictate material function. While initially pursued for optical enhancements, supramolecular design has since enabled the emergence of increasingly complex functionalities, including tunable selectivity and performance‐driven responsiveness. In this field, organic molecules can adopt distinct aggregate morphologies where morphology itself dictates photocatalytic selectivity, for example, to favor either hydrogen or hydrogen peroxide production, or enabling distinct long‐range charge transport pathways through controlled supramolecular packing, demonstrating how supramolecular organization directly control functional outcomes [24, 25, 26, 27].

These findings raise a broader, largely unexplored question: if self‐assembly pathways influence function, can function itself, such as catalytic activity, reshape the self‐assembly landscape? Could a catalytic process effectively “select” for the most active species, reinforcing its own persistence in a rudimentary form of chemical fitness?

Such feedback is a defining feature of natural systems and represents a foundational requirement for Darwinian‐like evolution in synthetic chemical environments, where differential fitness emerges from out‐of‐equilibrium dynamics [28, 29, 30]. Otto and co‐workers demonstrated this using minimal self‐replicators: photocatalytic cofactors enabled light‐driven protometabolism that reinforced the most active species and continuous irradiation ultimately selected replicators with superior catalytic function [31, 32].

**FIGURE 1:**
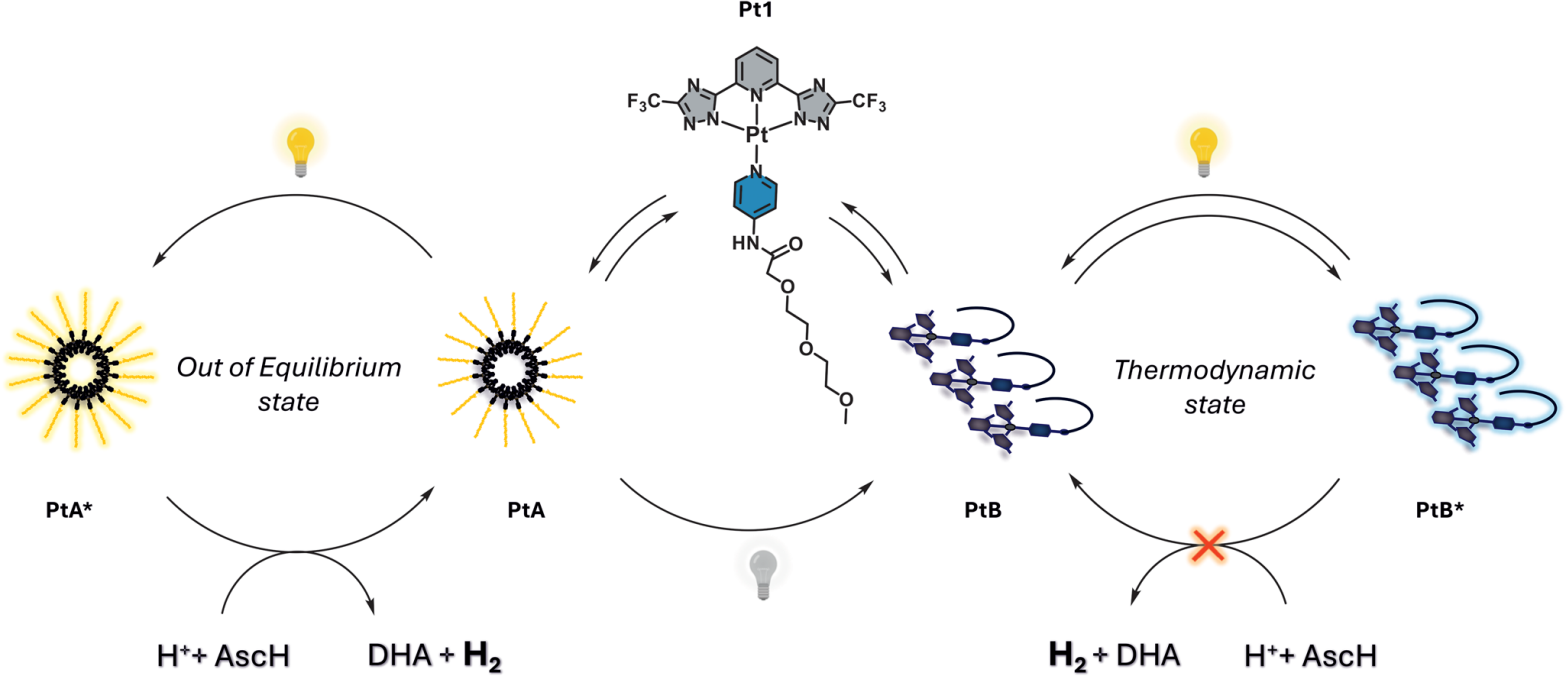
Catalytic‐dependent persistence of a self‐assembly system. The **Pt1** complex forms two aggregates: the active **PtA** state, sustained by light‐driven catalysis, and the inactive **PtB** state, which represents the thermodynamic endpoint. In acidic ascorbate buffer (AscH), **PtA** enables hydrogen evolution and dehydroascorbic acid (DHA) formation, while resisting thermodynamic conversion to **PtB**.

Here, we present a supramolecular system capable of both selecting the most functional species and allowing it to persist through external energy input, thereby preventing relaxation into its thermodynamic minimum (Figure 1). Specifically, platinum(II) complexes (**Pt1**) form two distinct emissive aggregates: (*i*) orange‐emissive particles (**PtA**), representing kinetically trapped structures stabilized by strong Pt···Pt interactions [33], and (*ii*) blue‐emissive 1D ribbons (**PtB**), the thermodynamic end state lacking significant metallophilic interactions [34]. Although **PtA** typically converts into **PtB** over time through solvent‐dependent kinetics [35], we discovered that **PtA** uniquely functions as both a visible‐light harvester and a hydrogen‐evolving catalyst in ascorbic acid aqueous solution operating without external photosensitizers or co‐catalysts. In stark contrast, **PtB** is catalytically inactive. In this work, we show that keeping **PtA** engaged in photocatalysis prevents its relaxation into the thermodynamic **PtB** state. This system effectively creates a minimal system where function dictates survival: energy consumption allows the persistence of a catalytically competent, far‐from‐equilibrium state.

## 2 Results and Discussion

**FIGURE 2:**
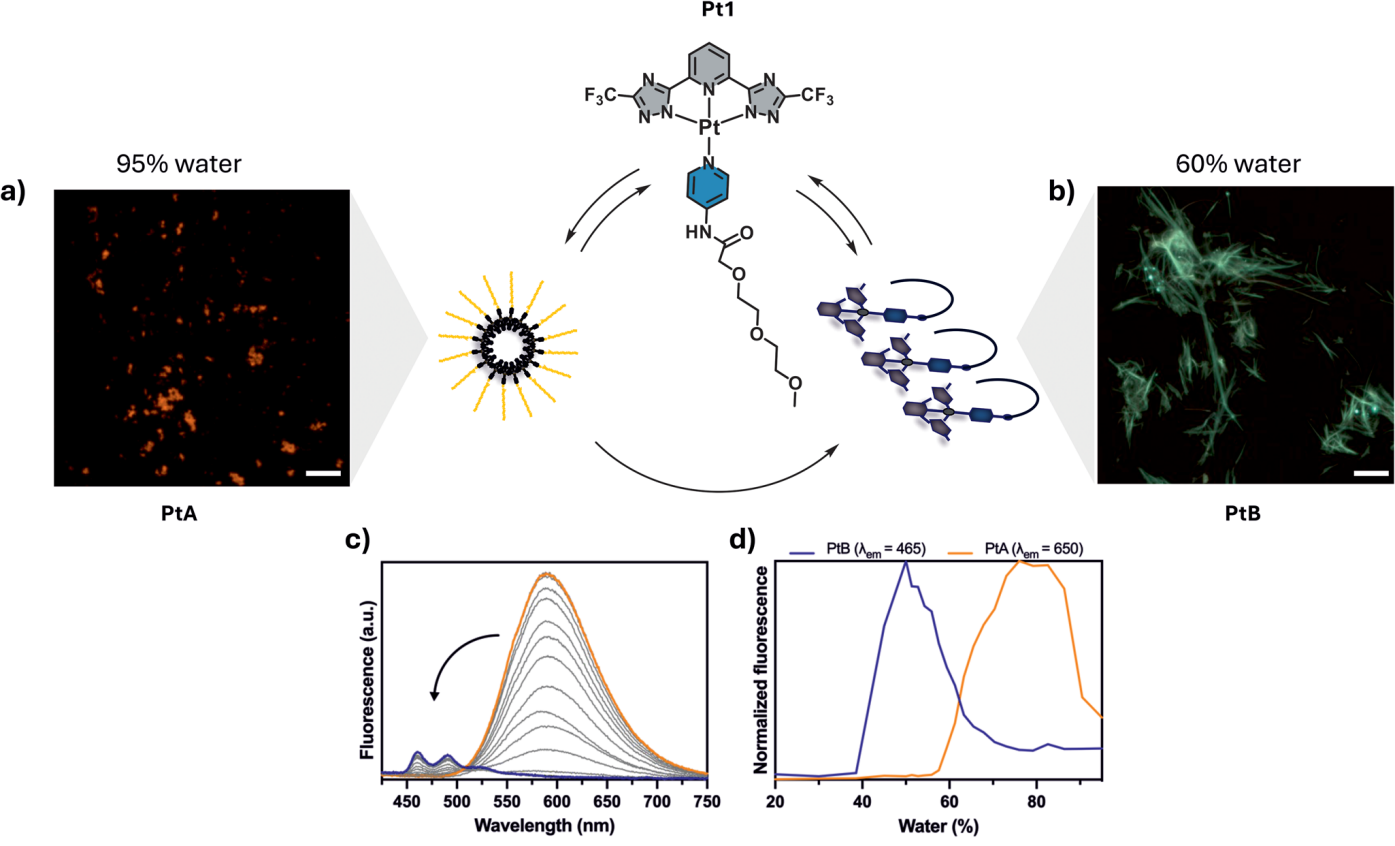
Photophysical and morphological characterization of **Pt1** self‐assembly. The **Pt1** complex forms two distinct supramolecular morphologies depending on solvent composition: micelle‐like **PtA** aggregates in water‐rich environments and fibril‐like **PtB** structures in more organic‐rich mixtures. (a) Fluorescence microscopy image of **PtA** formed in 95% water. The white rectangle indicates a 100 µm scale bar. (b) Fluorescence microscopy image of **PtB** formed in 60% water. The white rectangle indicates a 100 µm scale bar. (c) Emission spectra recorded at varying acetonitrile /water ratios (grey lines), showing a transition from **PtA** (orange line) to **PtB** (blue line); the black arrow indicates the direction of increasing acetonitrile content. (d) Normalized emission intensity at *λ*_em_ = 650 nm (**PtA**, orange) and *λ*_em_ = 465 nm (**PtB**, blue) plotted as a function of solvent composition.

Complex **Pt1** can adopt two distinct supramolecular morphologies: a kinetically trapped, micelle‐like aggregates (**PtA**) with close Pt···Pt interactions, and a thermodynamically stable, fibrillar structure (**PtB**) lacking metal–metal contacts, as confirmed by excitation spectra where the MMLCT band is absent (Figure S1) [34]. The triethylene glycol chain in the ancillary ligand plays a key role in defining these two morphologies. In **PtB**, the chain adopts a bent conformation due to intramolecular hydrogen bonding between the ether oxygens and the amino group, whereas in **PtA**, the chains extend outward toward into the polar solvent, stabilizing a micelle‐like morphology as previously established by Guillermo et al. where structural details are reported and just summarized here for completeness [36]. Solvent composition strongly dictates which morphology is favored. In a water‐rich environment (95% water/acetonitrile), **Pt1** assembled into kinetically trapped, orange‐emissive particles (**PtA**) (Figure 2a). The corresponding photoluminescence emission intensity at 600 nm gradually decreased as the acetonitrile content increased (Figure 2c,d). At 40% acetonitrile, the system transitioned into blue‐emissive fibrillar aggregates (**PtB**) (Figure 2b), as evidenced by the appearance of structured emission at 460 nm (Figure 2c). At 60% acetonitrile (v/v), **Pt1** remained fully monomeric and non‐emissive, indicating complete disassembly of the aggregates upon increasing the acetonitrile content (Figure 2d). As previously reported [34], this complex can spontaneously evolve toward the thermodynamically favored form, with the rate of transformation strongly influenced by the solvent environment. When the conversion is kinetically hindered, the process can be accelerated by bypassing the nucleation barrier. This is achieved by introducing preformed **PtB** fibrils as seeds into the **PtA** solution, which triggers a seeded growth pathway [35, 37]. These distinct supramolecular states provided us the foundation for exploring how morphology influences photocatalytic activity.

We knew from previous work that Pt(II)‐complexes are excellent catalysts for the hydrogen evolution reaction, especially when the Pt catalysts operate in tandem with a [Ru(bpy)_3_]^2+^ photosensitizer in multi‐component systems with methylviologen as electron relay and EDTA as sacrificial electron donor [38, 39, 40, 41]. Alternatively, the system could be a connected two multifunctional components in the form of a covalently bonded photosensitizer‐catalyst dyad [42, 43, 44, 45]. Simpler mononuclear Pt(II) complexes that possess both functionalities have been more recently reported [46, 47, 48, 49, 50, 51]. These Pt(II)‐only photocatalysts have been shown to evolve hydrogen within a single molecular motif in the only presence of a sacrificial electron donor.

We reasoned that supramolecular organization could provide an additional level of control, with the supramolecular morphology of **Pt1** influencing its performance in the photocatalytic hydrogen evolution. To test this, we evaluated and compared the potential hydrogen‐evolving activity of the three **Pt1** states. In a typical run, our catalytic mixture contained 100 µM total Pt content (as one of the three **Pt1** states), and 0.25 M ascorbic acid as sacrificial donor. The hydrogen production by the three **Pt1** states was evaluated by visible‐light irradiation using a 450 nm light‐emitting diode (LED, 200 mW∙cm^−2^). After irradiation, the headspace was sampled and analyzed by gas chromatography (GC) equipped with both a thermal conductivity detector (TCD) and a flame ionization detector (FID); further experimental details are provided in the Supporting Information.

**FIGURE 3:**
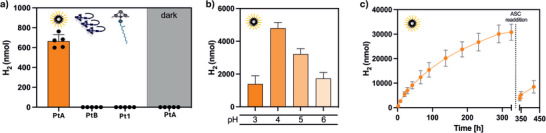
Characterization of aggregation‐induced photocatalysts. (a) Hydrogen production from different Pt morphologies (100 µM total Pt content, 0.25 M buffer ascorbate, pH 4, 2 h under blue light irradiation): PtA (95% water), PtB (60% water), monomeric Pt1 (40% water), and a control sample of PtA (95% water) in the dark. (b) Hydrogen production at different pH values at 100 µM total Pt content, 0.25 M buffer ascorbate, 24 h under blue light irradiation. (c) Kinetics of hydrogen production from PtA assemblies; the break in the *x*‐axis indicates the time point when additional sacrificial ascorbate was added.

Irradiation for 2 h of the mixture containing kinetically trapped **PtA** aggregates in aqueous ascorbate buffer produced 665 ± 66 nmol of hydrogen (Figure 3a). We have improved the **PtA**‐based hydrogen evolution system by adjusting the pH of the reaction mixture. The catalytic activity was dependent on the pH in the range between 3 and 6, with the hydrogen production of 649 ± 22 nmol after 24 h observed at pH 4 (Figure 3b). Thus, our **PtA** integrates both the photosensitizer and catalyst functions evolving hydrogen within a single molecular motif in the only presence of a sacrificial electron donor, which offers a clear advantage over previously reported multicomponent systems. No significant reaction was instead observed when we use monomeric **Pt1** under identical reaction (Figure 3a). Intriguingly, hydrogen production did not occur to any significant extent when **PtB** structures were used under the same conditions (Figure 3a). Likewise, **Pt1** and **PtB** remained inactive when irradiated with broadband white light or a 365‐nm LED to better match their absorption profile. To decouple size effects from intrinsic photocatalytic activity, the size of **PtB** aggregates was deliberately reduced by sonication and vortex mixing, yielding smaller **PtB** fragments as confirmed by optical microscopy (Figure S2). Photocatalytic experiments performed under identical conditions to those used for **PtA**, including the same solvent composition (95% water), still showed no detectable hydrogen evolution, indicating that the inactivity of **PtB** does not arise from aggregate size but from intrinsic differences in supramolecular structure and photophysical properties.

The difference in reactivity can be rationalized by metallophilic interactions. As extensively discussed elsewhere by others, Pt(II)_2_ dimers exhibit higher hydrogen‐evolving activities than mononuclear Pt(II) complexes, especially when dimeric compounds feature short Pt─Pt distance and/or strong metallophilic interactions [41]. We therefore attribute the inactivity of **PtB** to the absence of such interactions, which limits destabilization of its electronic structure. In **PtA**, close Pt···Pt contacts induce significant HOMO destabilization, raising its energy and lowering the oxidation potential [52]. By contrast, **PtB** lacks such these metallophilic interactions, resulting in a more stabilized HOMO at lower energy. Thus, controlling the supramolecular morphology of the **Pt1** complex enables the effective development of a Pt(II)‐based single‐molecular photocatalyst for hydrogen evolution, going beyond most prior efforts focused on controlling structural changes in the ligand system to enhance the hydrogen‐evolving activity.

Electrochemical studies corroborate these observations. Cyclic voltammetry of molecularly dissolved species **Pt1**, which lacks metallophilic interactions, can be used to estimate the oxidation potential of **PtB** aggregates. The cyclic voltammogram revealed two distinct oxidation processes: a dominant irreversible peak at +1.8 V, attributed to **PtB** aggregates, and a weaker peak at +1.1 V, assigned to **PtA** (Figure S3; see ESI for experimental details). The latter assignment is consistent with partial formation of **PtA** aggregates upon dissolution of **Pt1** in acetonitrile at 1 mM concentration. To confirm these assignments, we employed electrochemiluminescence (ECL) [52]. When Pt aggregates are exposed to oxidative potentials in the presence of a reducing agent like sodium oxalate, emission was detected only for **PtA** at +1.1 V, while **PtB** remained inactive (Figure S4). Similarly, when a reducing potential is applied in presence of PhICl_2_ as oxidating agent, light output was observed at +1.1 V, thus confirming **PtA** as the species responsible for the corresponding oxidation peak in the CV analysis, while the ECL signal in the reduction setup was at ‐1.1 V.

From the emission spectra (Figure S1), we extracted the E_0,0_ potentials of **PtA** and **PtB** as 2.48 eV (500 nm) and 2.75 eV (450 nm), respectively. Using these values, the excited‐state oxidation potential (E_ox_^*^) were estimated with Equation 1 at ‐1.38 for **PtA** ^*^ and ‐0.99 V for **PtB** ^*^, highlighting the stronger reducing character of PtA*. Conversely, the excited‐state reduction potential (E_red_^*^ derived from Equation 2) were 1.38 and 1.45 V, showing that PtB^*^ is the stronger oxidant.

(1) $$E_{\mathrm{ox}}^{*}=E_{\mathrm{ox}}-E_{0,0}$$

(2) $$E_{\mathrm{Red}}^{*}=E_{\mathrm{Red}}+E_{0,0}$$

The reduction potential of the Pt(II)* states is sufficiently more positive than the oxidation potential of the ascorbate (0.46 V) [53], making reductive quenching by ascorbate thermodynamically feasible and consistent with previous literature. Under these conditions, the reduced photocatalyst further undergoes a photoinduced step that drives hydrogen evolution from water, thereby regenerating the active Pt(II) species. In the absence of light, photocatalyst or sacrificial donor, the hydrogen evolution is negligible, confirming the photocatalytic nature of the process and the necessity of each component for the catalytic activity (Figure S5).

After prolonged light exposure, **PtA** exhibits remarkable long‐term stability and robustness, confirming its potential as a durable photocatalyst. UV–vis spectra showed negligible changes after 24 h of continuous irradiation, indicating structural integrity of the catalyst (Figure S6). The **PtA** photocatalyst remained productive over a period of over 300 h of continuous illumination achieving an overall hydrogen production of 30830 ± 3311 nmol (Figure 3c). Recovery of the initial activity is observed by re‐addition of the initial amount of sacrificial donor and further irradiation of the system (Figure 3c), thus proving the stability and longevity of the **PtA** photocatalyst.

The kinetically trapped orange‐emissive aggregates (**PtA**) slowly and spontaneously converts into the thermodynamically stable blue fibrils (**PtB**), a transformation that can be accelerated by triggering a seeded growth pathway [35]. The magnitude of the kinetic barrier is strongly solvent‐dependent [34]. In a more water‐rich environment (90% water), where the thermodynamic state transition energy barrier is significantly higher, **PtA** remains stable for more than 48 h even in the presence of **PtB** seeds, whereas, in a 70% water/30% acetonitrile solution, the addition of **PtB** “seeds” markedly accelerates the **PtA** → **PtB** conversion, which is complete within 15 min (Figure S7). This equilibrium‐like behavior shifts dramatically when the system is subjected to a functional task. Photocatalysis can act as a dynamic perturbation of the thermodynamic states, directing the supramolecular self‐assembly of the Pt complex, and providing a compelling framework for studying of chemical equilibrium, reminiscent of the classical Le Châtelier's principle.

**FIGURE 4:**
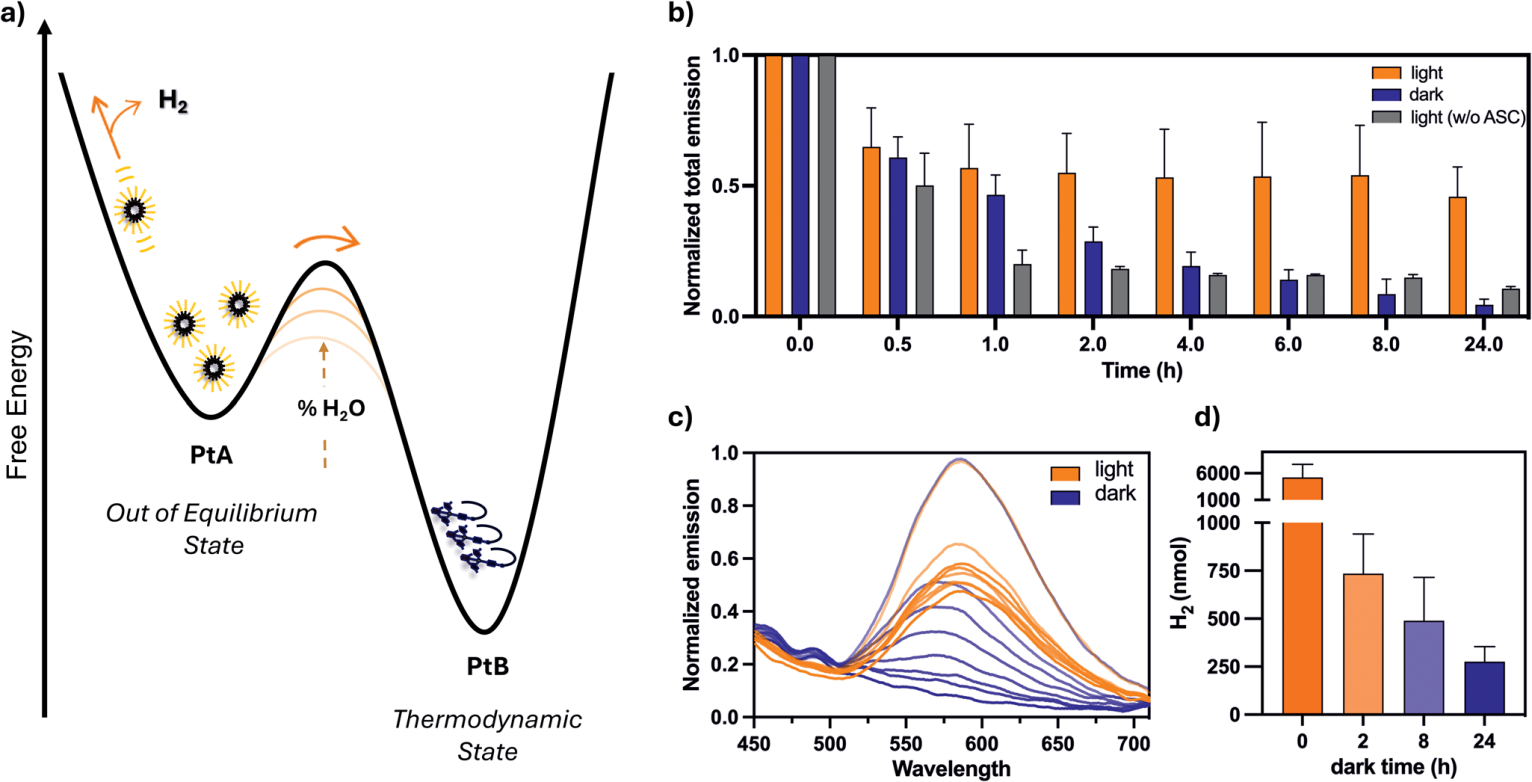
Out‐of‐equilibrium evaluation of the photocatalyst. (a) Energy diagram of the Pt assemblies: **PtA** represents a kinetically trapped state, while **PtB** corresponds to the thermodynamic state. The energy barrier between these states is modulated by the water content, and light is required to maintain **PtA** out of equilibrium. (b) Normalized total emission intensity vs time for a PtA (40 µM) and PtB (60 µM) mixture under dark and light conditions, in pH 4 buffer with 0.25 M ascorbate (85% water/acetonitrile), and under light irradiation without buffer ascorbate. (c) Emission spectra recorded during the kinetic evaluation: under photocatalytic condition, **PtA** retains its orange emission, while in the dark it transitions to the thermodynamic form. (d) Hydrogen production after 24 h of blue light irradiation for different systems (**PtA** 40 µM and **PtB** 60 µM, pH 4, 0.25 M ascorbate, 85% water/acetonitrile) following varying dark equilibration times before irradiation.

The interplay between kinetically trapped and thermodynamically favored states allows us to track how external stimuli, such as photocatalysis, drive the system out of equilibrium, modulate its structural organization, and, ultimately, control its functional output. During light‐driven hydrogen evolution, **PtA** enters a dissipative, out‐of‐equilibrium cycle: continuous photoexcitation keeps it far from its thermodynamic equilibrium, significantly prolonging its lifetime (Figure 4a). **PtA** persisted after 24 h of blue‐light irradiation in 85% H_2_O suspensions containing 40 µM **PtA** and 60 µM **PtB**, while the same **PtA**/**PtB** mixture kept in the dark or irradiated but in the absence of sacrificial electron donor at the same pH transitioned to the thermodynamically favored blue‐emissive **PtB** aggregate (Figure S8). Remarkably, even in the presence of pre‐formed **PtB** seeds, photocatalytic turnover preserved the orange‐emissive **PtA** state, underscoring the stabilizing effect of light‐driven reactivity. The kinetics of this behavior were monitored by tracking the emission spectra over 24 h, with quantification based on integration of the total emission band (Figure 4b,c). The remarkable feature of our system lies in its reliance on continuous light driven catalysis. **PtA** survives as long as photocatalysis remains active, but once irradiation ceases, it begins to decay toward its thermodynamic state. To probe this dynamic, we assessed the photocatalytic performance of identical **PtA**/**PtB** solutions after varying periods of dark pre‐incubation (0, 2, 8, and 24 h), followed by 24 h of irradiation. A clear trend emerged: the longer **PtA**/**PtB** solutions remained in the dark, the lower their subsequent hydrogen evolution activity (Figure 4d). The decline directly reflects the gradual transformation of the active **PtA** aggregates into the thermodynamically stable but inactive **PtB** form. Continuous photocatalytic turnover kinetically stabilizes the functional, out‐of‐equilibrium state, while darkness allows the system to drift back to its thermodynamic minimum, a change that is irreversible, as this equilibrium form cannot revert to the kinetically trapped state. This sharp contrast between active preservation and passive decay is vividly visualized by fluorescence microscopy after 24 h under light (Figure S9 right) and dark (Figure S9 left) conditions.

## 3 Conclusion

This work demonstrates how a synthetic supramolecular system based on platinum(II) complexes can operate under nature‐inspired, out‐of‐equilibrium conditions by coupling energy input to structural persistence. The kinetically trapped, orange‐emissive **PtA** aggregates—stabilized by metallophilic Pt···Pt interactions—act as efficient single‐component photocatalysts for hydrogen evolution, whereas the thermodynamically stable blue‐emissive **PtB** fibrils remain catalytically inactive.

Continuous irradiation sustains the **PtA** state by linking energy input to catalytic turnover, thereby preventing relaxation to the thermodynamic **PtB** form. Unlike many dissipative assemblies that can be regenerated upon refueling, this transition is irreversible: once energy input stops, **PtA** collapses into **PtB** and cannot be re‐formed.

This one‐way pathway creates a supramolecular scenario in which function—here, photocatalytic activity—reshapes the assembly landscape, selectively stabilizing the structure that remains active under sustained energy input. In doing so, the system provides a minimal, controllable platform for exploring how catalytic function and energy dissipation can govern persistence and structural selection in synthetic out‐of‐equilibrium assemblies.

## Conflicts of Interest

The authors declare no conflicts of interest.

## Supporting information




**Supporting File 1**: anie71786‐sup‐0001‐SuppMat.docx.

## Data Availability

The data that supports the findings of this study are available in the supporting information of this article
