# Supplementary material for: Out‐of‐Equilibrium Supramolecular Assembly Sustained by Photocatalysis
Source: Angew Chem Int Ed Engl. 2026 Mar 10;65(20):e9082267. doi: 10.1002/anie.9082267 (PMC13159418; doi:10.1002/anie.9082267)
Supplement: Supplementary file 1 — Supporting File 1: anie71786‐sup‐0001‐SuppMat.docx. [file ANIE-65-e9082267-s001.docx]

**Supporting Information**

**Out-of-equilibrium Supramolecular Assembly sustained by Photocatalysis**

E. Pelorosso^‡^, M. Scaccaglia^‡^, A. Fortunato, D. Alessi, F. Arcudi*, A. Aliprandi*

Dipartimento di Scienze Chimiche, Università degli Studi di Padova; Via Marzolo 1, Padova, 35131, Italy.

^‡^These authors contribute equally

*corresponding author: alessandro.aliprandi@unipd.it, francesca.arcudi@unipd.it

[1. Methods 2](#_Toc208305759)

[General 2](#_Toc208305760)

[Samples Preparation 2](#_Toc208305761)

[Hydrogen production 2](#_Toc208305762)

[Photophysical Measurements 3](#_Toc208305763)

[Electrochemical Measurements 3](#_Toc208305764)

[Kinetics of Morphological Conversion (PtA → PtB) 3](#_Toc208305765)

[Competitive Hydrogen Production vs. Morphological Conversion 4](#_Toc208305766)

[2. Supporting Figures 4](#_Toc208305767)

[3. Bibliography 7](#_Toc208305768)

# Methods

## General

**Pt1** was synthesized according to a previously reported procedure, and the ¹H NMR spectrum matched the previously reported characterization (*Isr. J. Chem.* **2019**, 59, 892–897).

^1^H NMR (CDCl_3_, 400 MHz, ppm) δ: 3.50 (s, 3H, CH_3_), 3.66-3.82 (m, CH_2_CH_2_-O-CH_2_CH_2_-O), 4.19 (s, 2H, CH_2_CO), 7.84 (d, ^3^*J_H_*_H_=7.7 Hz, 2H, Ar-H), 8.98-8.06 (m, 3H, Ar-H), 9.58 (br d, ^3^*J_H_*_H_=7.2 Hz, 2H, Ar-H), 9.58 (br s, 1H, N-H).

##

## Samples Preparation

- Stock Solution (**Pt1**): Dissolve **Pt1** in ACN (2 mM) by heating at 50 °C until fully dissolved.
- Kinetically Trapped Aggregate (**PtA**): Prepare by flash-injecting 100 µL of 2 mM **Pt1** in ACN into 1.9 mL distilled water (final: 100 µM Pt, 95:5 H₂O/ACN).
- Thermodynamic Aggregate (**PtB**):
  - For 100 µM **PtB**: Inject 100 µL of 2 mM **Pt1** into 1.2 mL H₂O + 0.7 mL ACN (final solution 60:40 H₂O/ACN).
  - For 0.8 mM **PtB**: Inject 0.8 mL of 2 mM **Pt1** into 1.2 mL H₂O.
  - Aggregation completes within 1 hour.
- Monomeric **Pt1**: Studied in 60:40 H₂O/ACN (non-aggregating condition).

## Hydrogen production

**Buffer Solution Preparation**: A 1 M sodium buffer ascorbate was prepared by dissolving appropriate ratios of sodium ascorbate and ascorbic acid in 25 mL of H_2_O to achieve specific pH values:

| **pH** | **Ascorbic Acid (g)** | **Na Ascorbate (g)** |
| --- | --- | --- |
| 3 | 4.12 | 0.317 |
| 4 | 1.98 | 2.64 |
| 5 | 0.564 | 4.318 |
| 6 | 0.064 | 4.88 |

The pH was adjusted using 1 M HCl or 1 M NaOH, and verified with a calibrated pH meter.

**Photocatalytic reactions**: Samples were prepared in 9.0 mL screw-cap vials (Thermo Scientific B7800-3) with micro stir bars (VWR 2×7 mm), sealed with silicone/PTFE septa (TS12713) and open-top caps (TS13216). Each vial contains 2 mL of 0.1 mM **Pt1** complex, 0.25 M buffer ascorbate, and a defined H_2_O/ACN ratio optimized for aggregate morphology. The vials were sealed and purged with N₂ for 15 minutes using steel needles inserted through the septum as inlet (inserted into the solution) and outlet (venting the headspace to the surrounding atmosphere). After purging for the time specified, the pressure of the headspace was then equilibrated to 1 atm. The vials were then illuminated (irradiated area 1.767 cm^2^) using a homebuilt photoreactor made of 450 nm LEDs (High Power LED Star, LEDsupply.com) with a light intensity of 200 mW∙cm^−2^ (measured using an Optical Power Meter PM100D with Optical Sensor S120VC from Thorlabs). Each vial was suspended on top of a single LED, equipped with a lens, using a homebuilt sample holder. The vials were continuously stirred at 300 rpm during irradiation. Experiments were conducted at least in triplicate; error bars represent standard error of the mean.

For kinetic H_2_-evolution measurements, the samples were periodically analyzed by gas chromatography (GC). After each sampling, the vials were returned to the irradiation setup and kept covered with modeling clay and Parafilm to minimize any potential exposure to air. GC analyses confirmed that no significant air exposure occurred during the experiments.

**Gas chromatography:** GC analyses were performed on an Agilent 8860 system with a PAL3 autosampler coupled with TCD detector at 250 °C. The GC was equipped with a molecular sieve columns. For hydrogen detection, the carrier gas was Argon at 6 mL/min and 14 psi. The oven temperature was kept constant at 100° C for 6 min (total run time 6 min). Headspace injections (100 µL) were performed using a PAL3 Autosampler with a gas-tight SGE syringe.

## Photophysical Measurements

**UV-Vis Absorption**: Recorded on a Varian Cary 100Bio UV-Vis spectrophotometer using 10 mm quartz cuvettes.

**Photoluminescence (PL) Spectra**: Acquired with an Edinburgh Instruments FLS1000 spectrometer (450 W Xe lamp, Hamamatsu R13456 detector). Solution samples were measured in 10 mm cuvettes.

**PL Quantum Yields**: Measured using a Hamamatsu Quantaurus QY (C11347) integrating sphere under air-equilibrated conditions with an empty quartz tube as reference.

**Fluorescence Microscopy**

Morphological changes were observed in real-time using a Zeiss Axio Observer 7 fluorescence microscope. Samples were excited at 385 nm (Light Source Colibri 5 Type RGB-UV; Wavelength Range: UV 385 ± 15 nm, blue 469 ± 19 nm, green 555 ± 15 nm, 631 ± 16.5 nm).

## Electrochemical Measurements

**Cyclic Voltammetry (CV)**

CV was performed on 1 mM Pt1 in ACN with 0.1 M TBAPF₆ as the supporting electrolyte, using an Ag wire as reference electrode. For comparison with literature values vs. Ag/AgCl, the Fc⁺/Fc potential was measured at –0.47 V and corrected by +0.45 V. The potential was swept 0 → 2 V → –2 V → 0 V at a scan rate of 0.1 V/s.

**Electrochemiluminescence (ECL)**

ECL measurements were conducted on:

- 1 mM **Pt1** in DMF/H₂O (2:1) with 0.1 M TBAPF₆, and 5 mM sodium ascorbate (pH 13). CV was applied (0 to 2 V vs. Ag/AgCl, 0.02V/s), and emission was monitored via a nearby PMT.
- **PtA** was precipitated in 75:25 ACN/H₂O to 0.5 mM final Pt, using KClO₄ as electrolyte and 10 mM sodium oxalate. Another identical sample was equilibrated to form **PtB** prior to analysis.
- 1 mM **Pt1** in DMF/H_2_O (1:1) with 0.1M KClO_4_ and 0.1M PhICl_2_. CV was applied (0 to -2 V vs. Ag/AgCl, 0.02V/s), and emission was monitored via a nearby PMT.

## Kinetics of Morphological Conversion (PtA → PtB)

A series of vials containing fixed buffer ascorbate (0.25 M) and varying H_2_O/ACN ratios were prepared. Mixtures contained 60 µM **PtB** and 40 µM **PtA** (total [Pt] = 0.1 mM). Control samples with only 40 µM **PtA** were also tested.

Samples were degassed and exposed to blue light irradiation, while controls were kept in the dark. Morphological changes were monitored visually under a UV lamp at time points: 0, 0.5, 1, 2, 3.5, 5.5, 8, 24, and 48 h. For the optimal conversion condition, emission intensity and quantum yield were quantified using an integrating sphere.

## Competitive Hydrogen Production vs. Morphological Conversion

A 40 mL batch containing **PtA** (40 µM), **PtB** (60 µM), 0.25 M buffer ascorbate, and 85% H_2_O was prepared and divided into multiple 2 mL vials. All samples were degassed as described. One set was immediately irradiated for 24 h. The remaining sets were equilibrated in the dark for 2, 8, or 24 h before 24 h light exposure. Hydrogen production was measured by GC as previously described.

# Supporting Figures

**
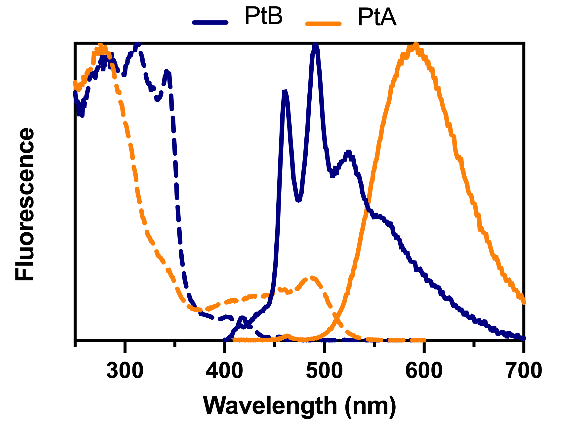
**

**Figure S1:** Excitation (dashed line, λ_em_=530 nm and 460 nm) and emission (solid line, λ_exc_=345 nm) in 60% (blue line) and at 95 % water content (orange line) for **Pt1**. The concentration is 50 μM.

| **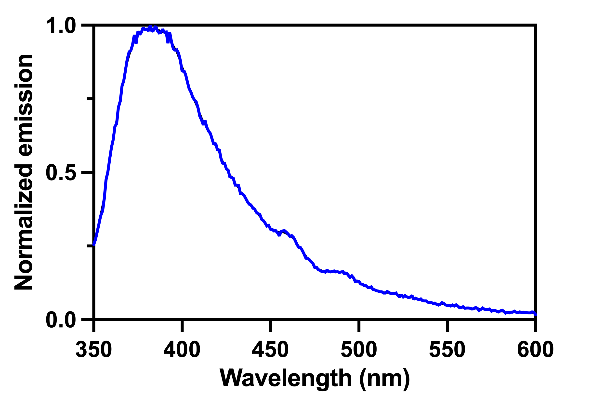** | **** |
| --- | --- |

**Figure S2**. Photocatalytic performance of size-reduced **PtB** aggregates, prepared to approximate the size of **PtA**, under identical solvent conditions (95% water). Left: emission spectra recorded after photocatalysis, confirming the exclusive presence of **PtB** and the absence of orange-emissive **PtA** species. Right: optical microscopy images of **PtB** aggregates after photocatalysis, showing fragmented fibrillar structures.


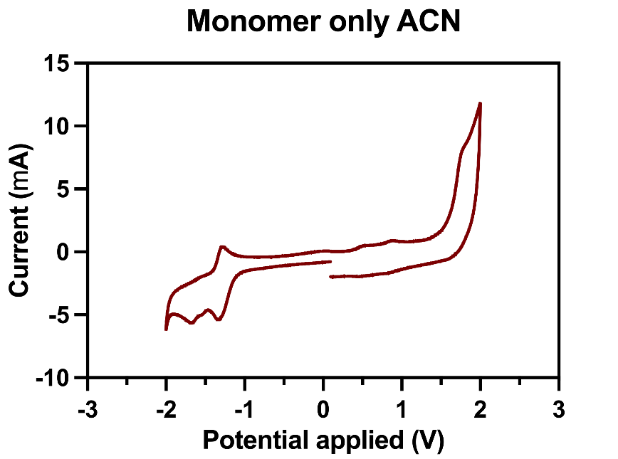


**Figure S3**: CV of **Pt1** (10^-3^ M in ACN) using TBAPF_6_ (0.1 M) as supporting electrolyte and Ag wire as reference electrode; the potential of ferrocene, used as standard, is 0.47 V. Applied potential ranging from 2 V to -2 V (Ox-Red cycle), scan rate 0.1 V/sec.


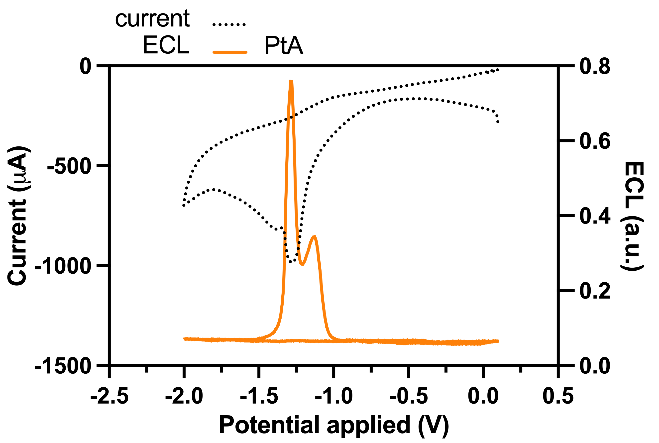

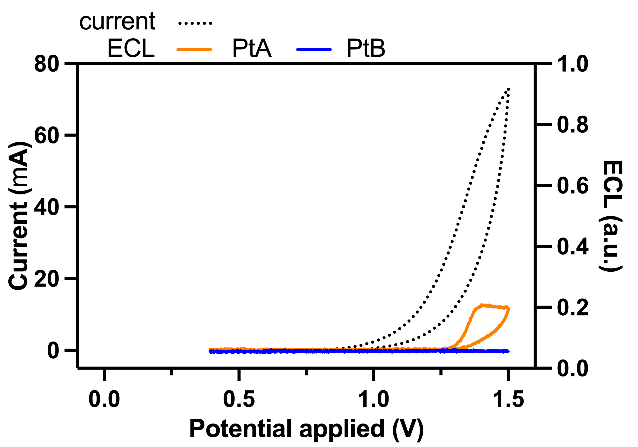


**Figure S4**: ECL intensity during a potential scan of 1 mM DMF/ H_2_O 1:1 solution of **Pt1** as **PtA** aggregate (orange trace) or **PtB** aggregate (blue trace) using 0.1 M KClO_4_ as supporting electrolyte, upon addition 10 mM of PhICl_2_ (left) and 10 mM sodium oxalate (right). Scan rate = 0.02 V s^–1^. GC as working electrode, Ag wire as reference electrode, the potential of ferrocene, used as standard, is 0.47 V, and Pt wire as counter electrode.


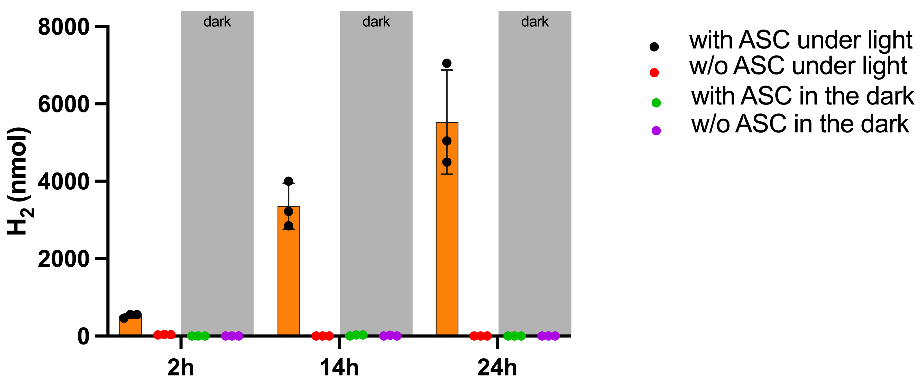


**Figure S5**: Time-dependent hydrogen production from **PtA** (40 μM) in 95% water under blue light irradiation or in the dark, in the presence or absence of the sacrificial electron donor ascorbate.


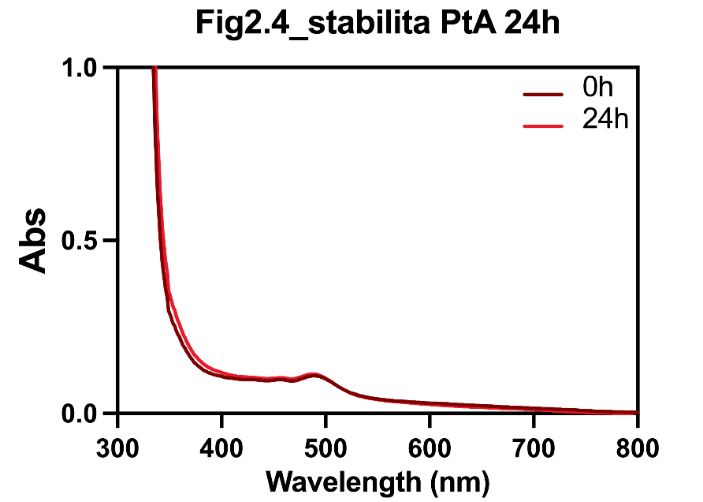


**Figure S6:** Absorption spectra of **PtA** at 95% water content, at 100 μM at time 0 and 24 h of irradiation (blue light at 465 nm).

**Figure S7**: Time-dependent morphological changes in a mixture of **PtA** (40 μM) and **PtB** (60 μM) with 0.25 M ascorbate at pH 4, under varying H_2_O/ACN (70–90%) and both dark and light conditions (left). A control experiment with only **PtA** (40 μM) under identical conditions serves as a seed-free kinetic reference (right).

**
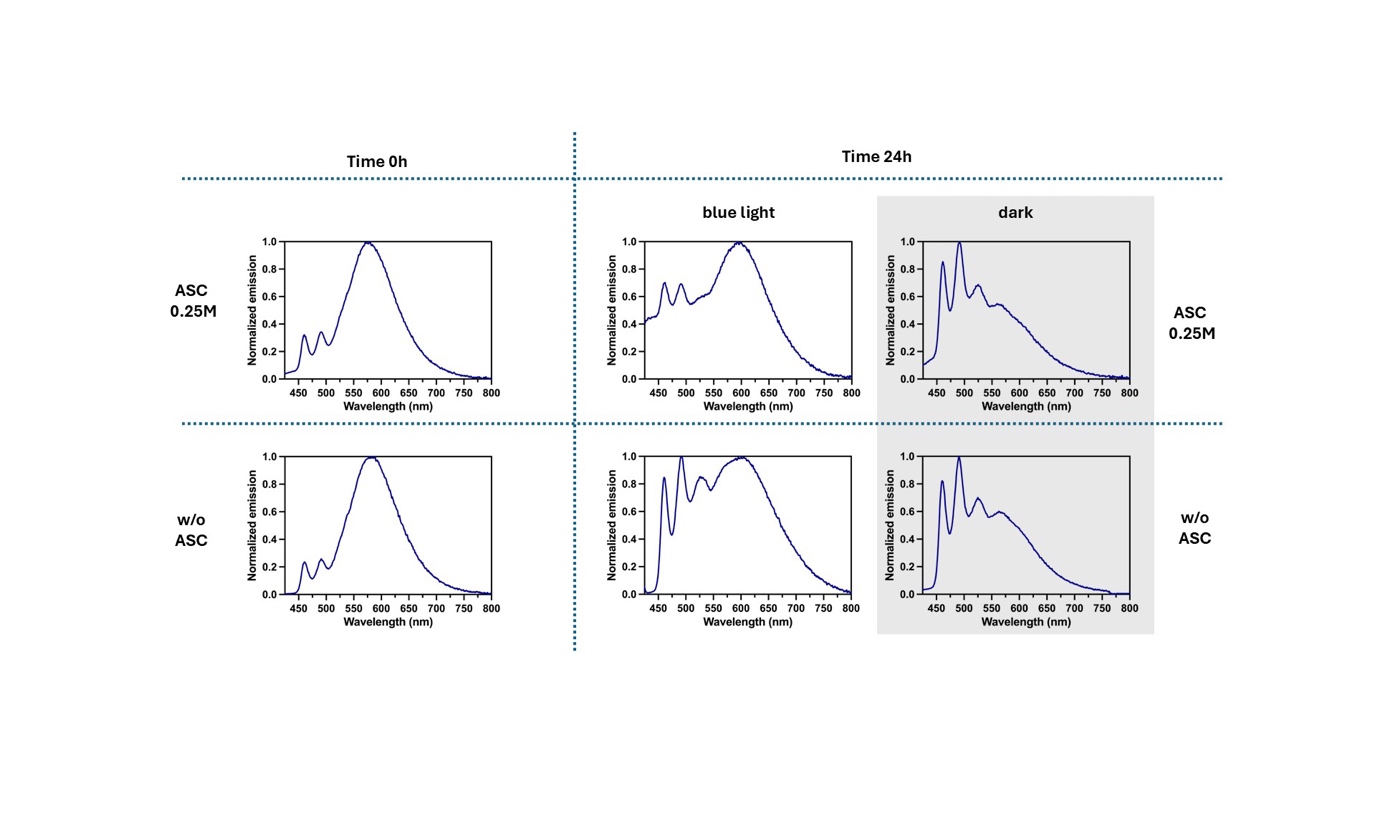
**

**Figure S8:** Emission spectra of a mixture of **PtA** (40 μM) and **PtB** (60 μM) in the presence (above) or absence (below) of 0.25 M ascorbate at pH 4, in 85% H₂O/ACN, under both light and dark (shaded) conditions, at time 0 and after 24 h.

| 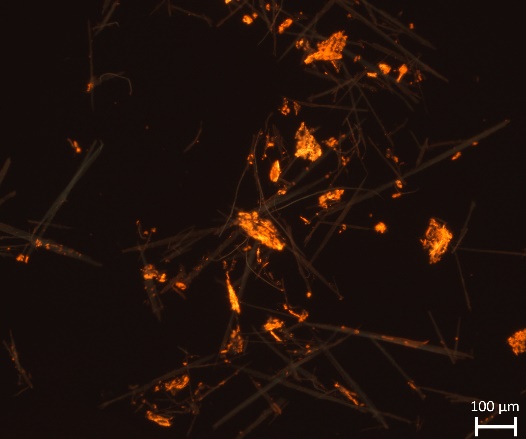 | 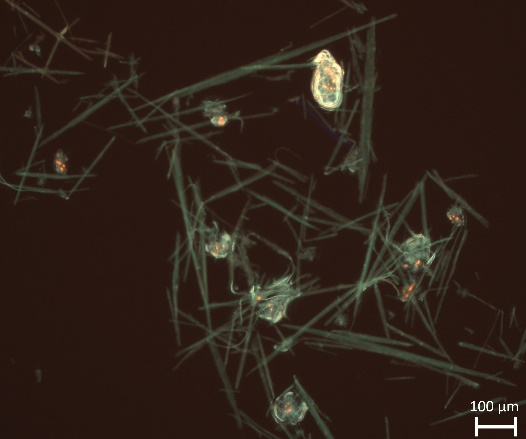 |
| --- | --- |

**Figure S9**: Morphological characterization at the fluorescent microscopy of the mixture system **PtA** 40 μM and **PtB** 60 μM, pH 4, 0.25 M ascorbate, 85% H_2_O/ACN after 24 h under photocatalytic conditions (left) or kept in the dark (right).
